# Aortopathy: limitations of diameter-based decision-making: a case report

**DOI:** 10.1093/ehjcr/ytag672

**Published:** 2026-09-07

**Authors:** Juliana Senftinger, Michael Stiefel, Robert Manka, Mark Haykowsky, David Niederseer

**Affiliations:** High Mountain Clinica Davos, Medicine-Campus, Herman-Burchard Strasse 1, 7265 Davos, Switzerland; Department of Cardiology, University Heart and Vascular Centre Hamburg-Eppendorf, Martinistraße 52, 20251 Hamburg, Germany; Department of Cardiology, University Hospital Zurich, Rämistrasse 100, 8091 Zurich, Switzerland; Department of Radiology, University Hospital Zurich, Rämistrasse 100, 8091 Zurich, Switzerland; High Mountain Clinica Davos, Medicine-Campus, Herman-Burchard Strasse 1, 7265 Davos, Switzerland; College of Health Sciences, Faculty of Nursing, University of Alberta, 116 85 Ave NW, AB T6G 2R3 Edmonton, Canada; High Mountain Clinica Davos, Medicine-Campus, Herman-Burchard Strasse 1, 7265 Davos, Switzerland; Center of Translational and Experimental Cardiology (CTEC), Department of Cardiology, University Heart Center Zurich, University Hospital Zurich, University of Zurich, Rämistrasse 100, 8091 Zurich, Switzerland; Christine Kühne-Center for Allergy Research and Education (CK-CARE), Medicine Campus Davos, Herman-Burchard Strasse 1, 7265 Davos, Switzerland

**Keywords:** Ascending thoracic aortic aneurysm (ATAA), Endurance athlete, Aortic wall stress, Aortic wall thickness, Shared decision-making, Case report

## Abstract

**Background:**

Thoracic aortic aneurysms are often incidental findings that require close follow-up monitoring and eventual surgical or endovascular treatment to prevent life-threatening dissection. Their complex relationship with physical activity requires further research to balance cardiovascular risk reduction against the dangers of acute blood pressure peaks.

**Case summary:**

A 75-year-old male endurance athlete was evaluated in a sports cardiology clinic to assess the safety of intensive exercise with a known ascending thoracic aortic aneurysm (ATAA). He had adopted an active lifestyle at 65 and completed over thirty 70.3 Ironman races. In 2019, at the age of 75, echocardiography revealed an ATAA measuring 52 mm, along with a bicuspid aortic valve and moderate regurgitation. The patient declined surgery and wished to continue endurance training. After shared decision-making, continued training and racing were allowed under predefined limits for blood pressure and heart rate, with regular imaging surveillance. Over 6 years of follow-up using magnetic resonance imaging, computed tomography, and echocardiography, aneurysm size remained stable, with an aortic wall thickness of 2.5 mm. Based on Laplace’s law, the calculated circumferential wall stress stayed below rupture thresholds during exercise. Exercise counselling should consider not only aneurysm diameter but also blood pressure, wall thickness, and wall stress.

**Discussion:**

In accordance with current guidelines, watchful waiting and a temporary restriction of physical activity are recommended in this case of aortic dilatation. However, because factors such as increased aortic wall thickness may allow for a more favourable distribution of wall stress, an individualized risk assessment can refine these recommendations. In the present case, a shared decision-making approach revealed that controlled physical exertion did not critically increase wall stress and potentially conferred a long-term protective effect against aneurysm progression, underscoring the value of individualized therapeutic strategies.

Learning points

- Aneurysm diameter alone may be insufficient for guiding endurance exercise recommendations in patients with ascending thoracic aortic aneurysms.
- In accordance with Laplace’s law, evaluating exercise-related risk requires the integration of all determinants of aortic wall stress, specifically systolic blood pressure, aortic diameter, and aortic wall thickness.
- Under close surveillance and shared decision-making, carefully selected patients may safely continue endurance exercise over the long term without evidence of aneurysm progression.

## Background

The prevalence of thoracic aortic aneurysms in men aged 60–75 years is ≤0.1%.^1^ In recent years, prevalence has fortunately been declining due to improved management of risk factors and preventive measures.^2^ In many cases, thoracic aortic aneurysms are incidental findings in asymptomatic patients. With increasing dilatation, there is a risk of life-threatening rupture or dissection, which is why close follow-up monitoring is required, and once a certain diameter is reached, surgical or endovascular treatment of the aneurysm is recommended.^3^

The relationship between aortic aneurysms and physical activity is complex and requires a comprehensive risk assessment. On the one hand, physical activity reduces cardiovascular risk and thus may lower the likelihood of developing an aneurysm.^4^ On the other hand, acute peaks in blood pressure can potentially trigger complications such as dissection or rupture. Further research is needed to define the optimal frequency and intensity of physical activity in this patient population.

## Summary figure

**Figure:**
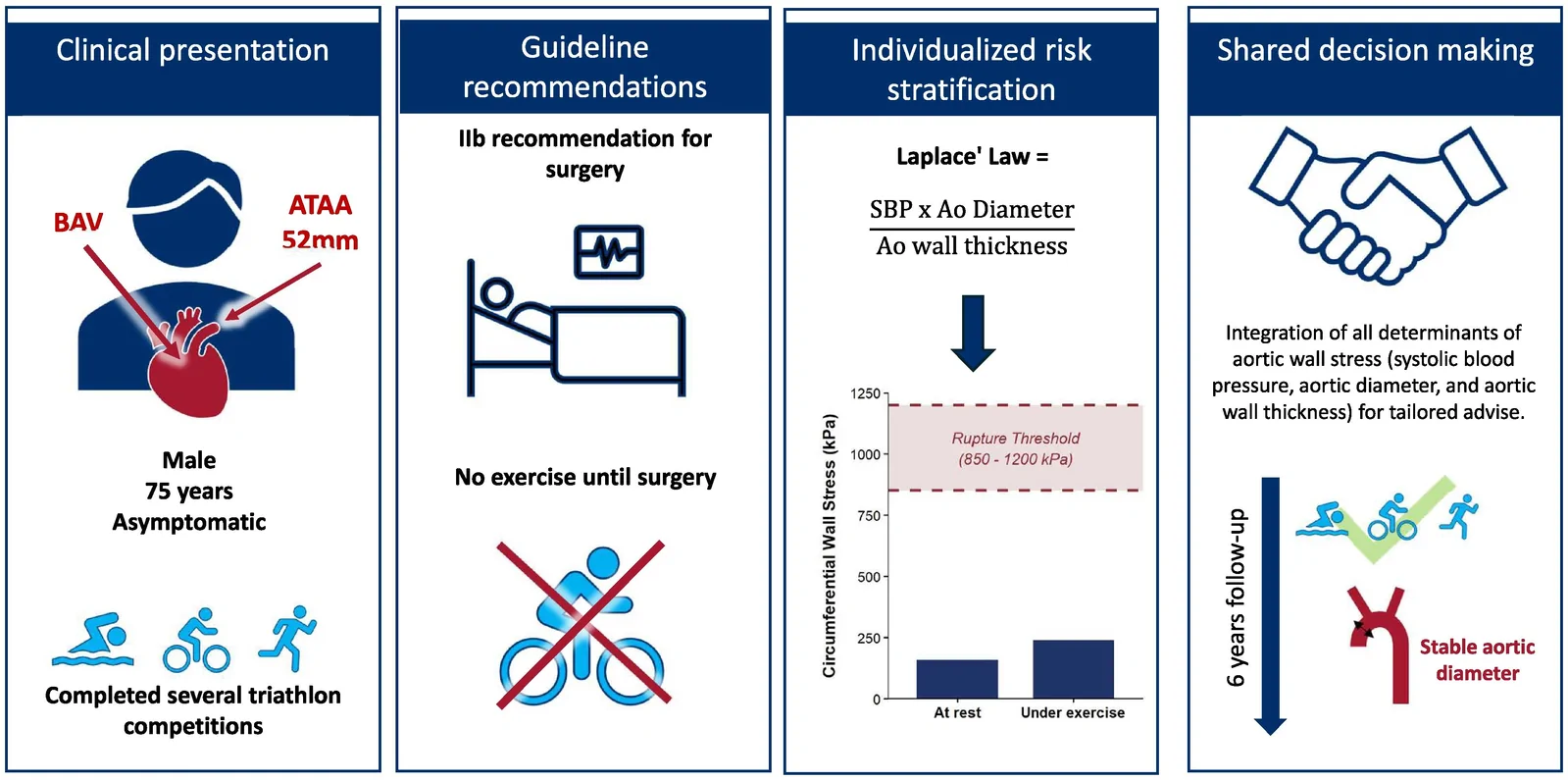


## Case presentation

A male individual in his 70s was initially evaluated in the sports cardiology outpatient clinic for therapeutic assessment and evaluation of the safety of engaging in intensive physical activity in the context of a known ascending aortic aneurysm.

The patient adopted a healthier lifestyle around the age of 65, initiating endurance training and completing over thirty 70.3 Ironman races (‘Half ironman’). He was 164 cm tall and weighed 67 kg, which remained stable during the follow-up. At the age of 75, a routine echocardiographic examination revealed an ascending thoracic aortic aneurysm (ATAA) measuring 52 mm in diameter.

**Figure 1:**
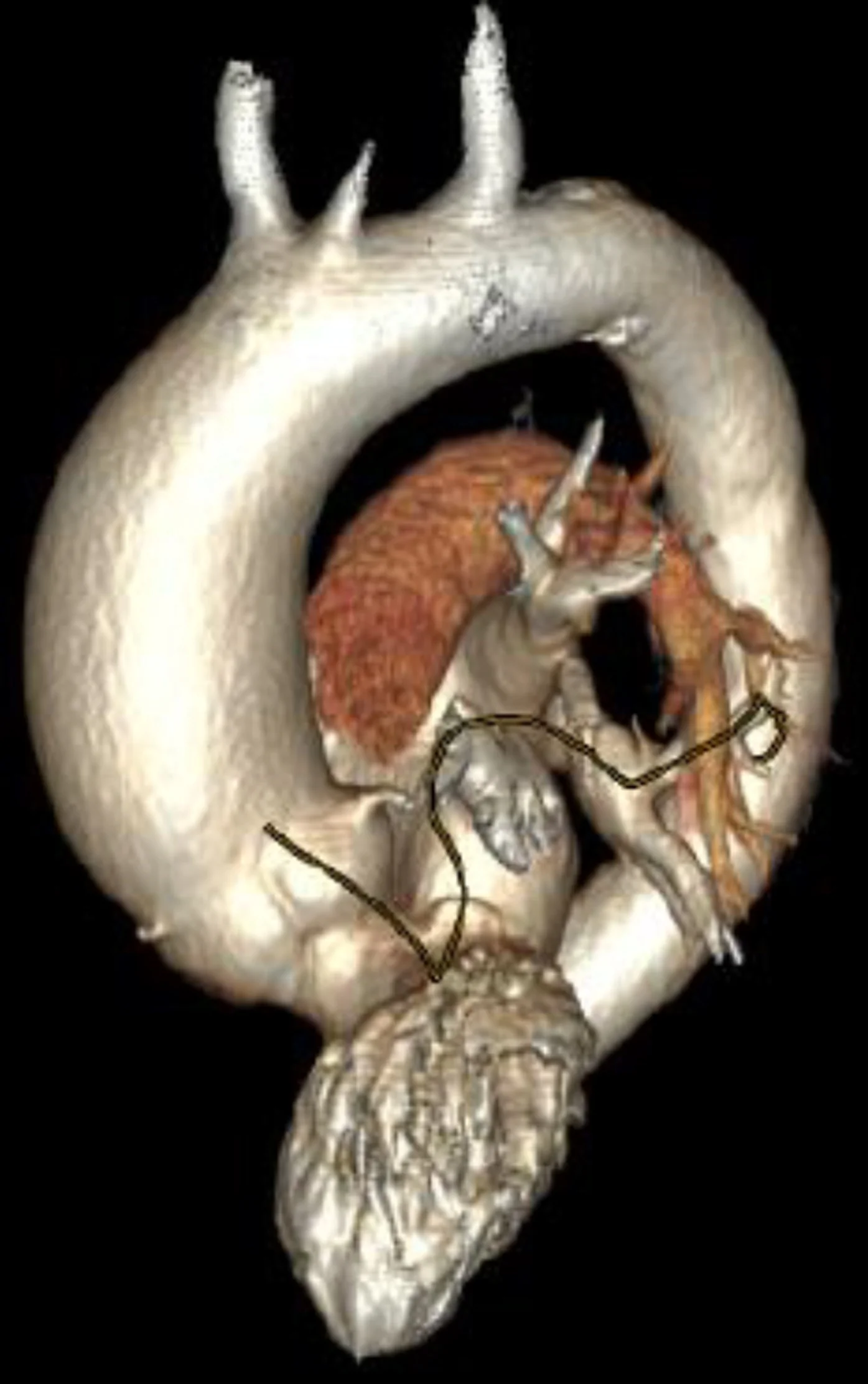
Computed tomography scan with a three-dimensional reconstruction of the thoracic aorta. Anulus 3.5 cm, sinus Valsalva 4.3 cm, sinutubular junction 4.0 cm, ascending aorta 5.2 cm, aortic arch after the origin of the left common carotid artery 3.0 cm, and descending aorta after the origin of the left subclavian artery 2.6 cm.

At the time of the initial presentation, the patient was asymptomatic, particularly with regard to cardiac symptoms. The aortic aneurysm diagnosed by echocardiography was confirmed by an additional computed tomography (CT) examination (*Figure 1*). Echocardiography further revealed a bicuspid aortic valve with fusion of the right and left coronary cusps, associated with moderate aortic regurgitation. The diameter of the aortic sinus measured 43 mm. With a maximum velocity of 40 cm/s in the descending aorta, there was no evidence of coarctation.^5^

Twenty-four-hour ambulatory blood pressure monitoring demonstrated an average blood pressure of 114/71 mmHg with a preserved nocturnal dipping pattern. Exercise testing revealed exercise-induced hypertension, with a maximum blood pressure of 238/119 mmHg. As an antihypertensive medication, the patient was taking bisoprolol, which also reduces heart rate and wall stress. In repeated cardiopulmonary exercise tests, his VO_2_ peak ranged between 21 and 27 mL/min/kg, well above the age-predicted values for his age. The patient also presented with hypercholesterolaemia as a cardiovascular risk factor. Consequently, lipid-lowering therapy with rosuvastatin had already been initiated, resulting in an LDL cholesterol level of 2.3 mmol/L. The patient did not take any performance-enhancing drugs or dietary supplements.

**Figure 2:**
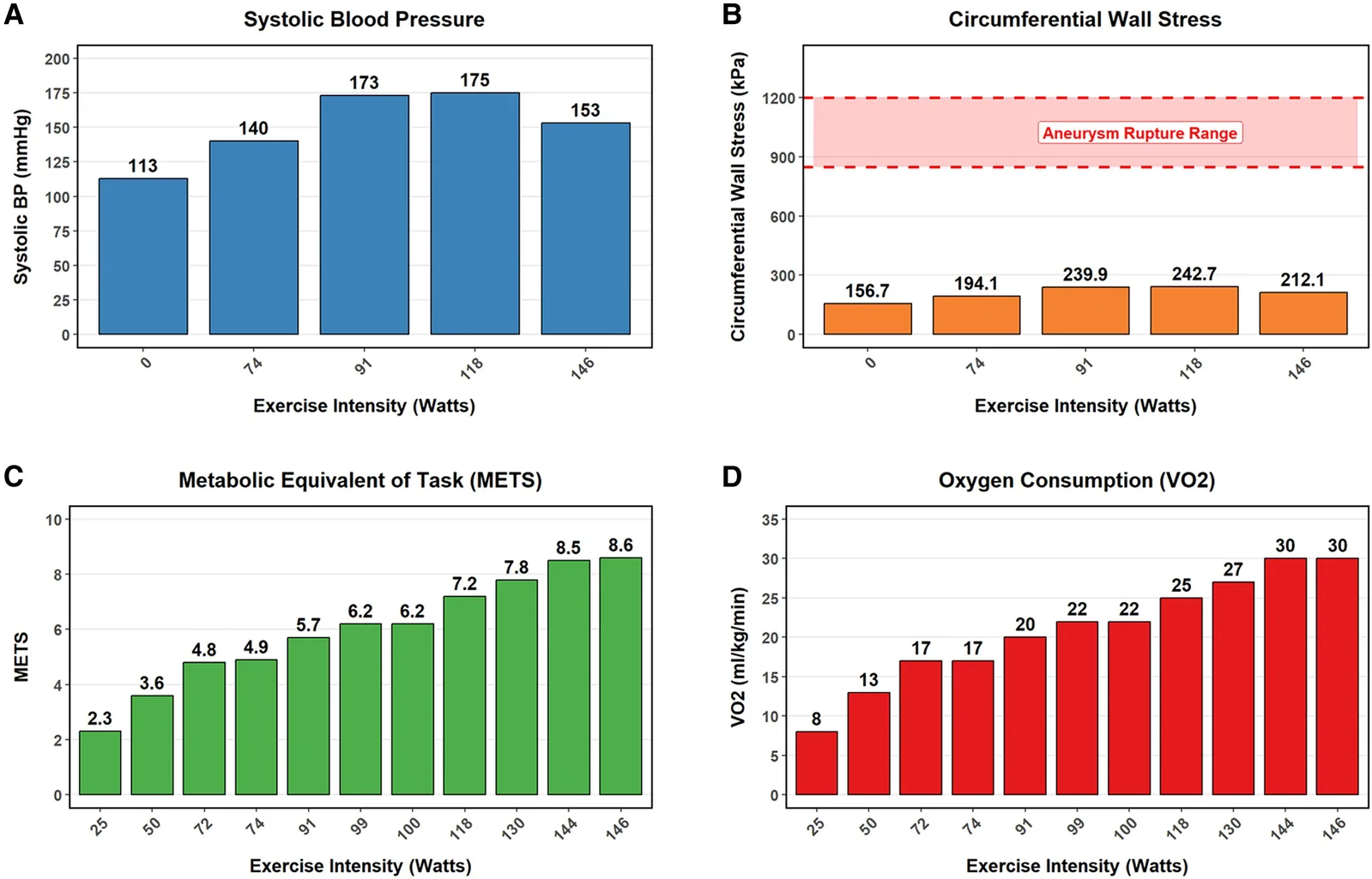
Haemodynamic and exercise response parameters at increasing exercise intensities. (*A*) systolic blood pressure (mmHg); (*B*) circumferential aortic wall stress (kPa) with the aneurysm rupture range indicated in red; (*C*): metabolic equivalents of task (METS); (*D*): oxygen consumption (VO_2_, mL/kg/min).

Cardiac magnetic resonance imaging (MRI) demonstrated an aortic wall thickness of 2.5 mm. Previous investigations have shown that aortic wall thickness and aortic diameter do not change under physical stress.^6^ Based on Laplace’s law circumferential aortic wall stress, equal to systolic blood pressure × aortic diameter/wall thickness, the calculated wall stress at rest was 157 kPa and increased to 243 kPa during maximal exercise, based on the values from the most recent cardiopulmonary exercise test in 2025 which are 52 mm for the aortic diameter, 2.5 mm for the wall thickness, a resting systolic blood pressure of 113 mmHg, and 175 mmHg at peak exercise (*Figure2*). These values are substantially below the wall stress thresholds associated with aneurysm rupture reported in the literature (850–1200 kPa).^7^

The patient declined surgical treatment and expressed the wish to continue his sports activity. Although the 2020 European Society of Cardiology Guidelines on sports cardiology and exercise in patients with cardiovascular disease do not recommend physical activity in this case until successful treatment is achieved, the situation can be reassessed afterwards.^8^

Following shared decision-making, several recommendations were made. First, the addition of an antihypertensive agent was suggested to reduce blood pressure peaks during physical activity. Furthermore, the patient was advised to limit exercise to a maximum heart rate of 120 beats/min, corresponding to a maximum blood pressure of 170/90 mmHg. In addition, participation in high-intensity training or contact sports was discouraged. Competitive events were selected in consultation with the treating sports cardiologist to avoid, for example, courses with steep climbs. Regular follow-up imaging was agreed upon, including echocardiography every 6 months, a CT scan after 2.5 years, and MRI examinations after 4.5 and 5.5 years.

During a 6-year follow-up, the ATAA remained stable, measuring 52 mm in the most recent cardiac MRI performed in June 2025. The patient continues to engage in regular exercise, in the context of shared decision-making under close sports cardiology supervision, with periodic imaging surveillance of the ascending aorta. Specifically, he exercises for 8–10 h/week, of which about 50% consists of endurance training (at mostly moderate intensity, 100–120 per min equivalent to 150–170 mmHg systolic blood pressure), and he continued to participate in triathlon events.

Following an ischaemic stroke in the territory of the left middle cerebral artery in June 2022, paroxysmal atrial fibrillation was detected using an event recorder. Consequently, a treatment with Lixiana (edoxaban) was initiated.

## Discussion include a very brief review of similar published cases

In accordance with current guidelines, in the absence of high-risk features—as the blood pressure was well controlled—a watchful waiting strategy with reimaging in 6 months was the appropriate recommendation despite an ascending aortic diameter of 52 mm.^3^ Furthermore, at least temporarily, abstinence from athletic activity is advised, with re-evaluation following successful treatment of the aneurysm.^8^ Smaller studies have suggested an association between acute aortic dissections and high-intensity physical activity, particularly weightlifting.^9^ Accordingly, current guidelines would not recommend vigorous physical activity in this patient, as outlined above.^8^ Mild aortic dilatation has been described in 20% older master athletes; however, pronounced forms are uncommon.^10,11^ In part, this mild enlargement reflects physiological aortic remodelling, with athletes demonstrating aortic root diameters approximately 3.2 mm larger at the level of the sinuses of Valsalva compared with sedentary controls. This finding is generally considered a normal adaptation to the haemodynamic stress of exercise rather than a pathological enlargement.^12^ The 2025 American Heart Association/American College of Cardiology scientific statement provides more specific guidance: Athletes with unexplained thoracic aortic dilatation ≤42 mm can generally participate in competitive sports, whereas those with diameters ≥45 mm are exposed to risks that likely outweigh the benefits.^13^ Although aortic diameter remains the strongest predictor of aortic dissection, nearly 60% of type A dissections occur in patients with diameters <5.5 cm, suggesting that additional factors should be taken into account.^14^ Retrospective analyses have demonstrated that ruptured aortic aneurysms are associated with reduced wall thickness.^15^ Conversely, increased wall thickness may allow for a more favourable distribution of wall stress and reduced peak wall stress, which also appears to be the case in our patient.^16^ To date, there are insufficient data on the impact of physical activity on aortic wall thickness. Further research in this area would be valuable to enable more individualized recommendations regarding physical activity in patients with aortic dilatation. This is particularly relevant given the well-established systemic benefits of exercise, including its favourable effects on arterial hypertension, which itself is a known risk factor for the progression of aortic aneurysms.^3^ In the context of shared decision-making, a simplified method could be applied by measuring aorta diameter and wall thickness from standard clinical CT and MRI scans, the most precise imaging modalities for this purpose, and estimating wall stress during physical activity using systolic blood pressure values recorded during ergometry, for example.^17^

In this case, we observed that under controlled conditions, physical exertion did not result in a clinically relevant increase in wall stress. Moreover, sustained physical activity may have conferred a potential long-term protective effect, as no progression in aortic diameter was noted during follow-up. This case underscores the importance of shared decision-making and highlights the value of individualized therapeutic strategies.

## Lead author biography


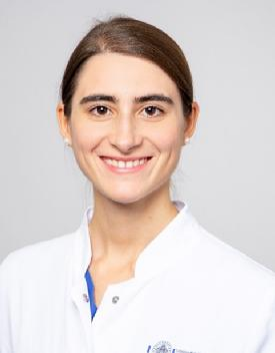


Dr. Juliana Senftinger is a cardiology resident at the University Heart and Vascular Center in Hamburg, Germany, who graduated from the University of Regensburg. She is currently working as a study physician at the Hochgebirgsklinik Davos as part of a scientific exchange programme. Her scientific work focuses primarily on acute coronary syndromes, advanced cardiovascular imaging, and the impact of lifestyle factors on cardiovascular health.

## Data Availability

The data underlying this article are available in the article.
